# Transcranial direct current stimulation for gait recovery following stroke: A systematic review of current literature and beyond

**DOI:** 10.3389/fneur.2022.953939

**Published:** 2022-09-07

**Authors:** Xavier Corominas-Teruel, Rosa María San Segundo Mozo, Montserrat Fibla Simó, Maria Teresa Colomina Fosch, Antoni Valero-Cabré

**Affiliations:** ^1^Department of Psychology, Neurobehavior and Health Research Group (NEUROLAB), Universitat Rovira i Virgili, Tarragona, Spain; ^2^Cerebral Dynamics, Plasticity and Rehabilitation Group, Institut du Cerveau et de la Moelle Epinière, CNRS UMR 7225, Paris, France; ^3^Rehabilitation and Physical Medicine Department, Hospital Universitari Joan XXIII, Tarragona, Spain; ^4^Cognitive Neuroscience and Information Tech. Research Program, Open University of Catalonia (UOC), Barcelona, Spain; ^5^Department of Anatomy and Neurobiology, Laboratory of Cerebral Dynamics, Boston University School of Medicine, Boston, MA, United States

**Keywords:** non-invasive brain stimulation (NIBS), transcranial direct cortical stimulation (tDCS), stroke, neurorehabilitation, neuromodulation, gait rehabilitation, biophysical modeling

## Abstract

**Background:**

Over the last decade, transcranial direct current stimulation (tDCS) has set promise contributing to post-stroke gait rehabilitation. Even so, results are still inconsistent due to low sample size, heterogeneity of samples, and tDCS design differences preventing comparability. Nonetheless, updated knowledge in post-stroke neurophysiology and stimulation technologies opens up opportunities to massively improve treatments.

**Objective:**

The current systematic review aims to summarize the current state-of-the-art on the effects of tDCS applied to stroke subjects for gait rehabilitation, discuss tDCS strategies factoring individual subject profiles, and highlight new promising strategies.

**Methods:**

MEDLINE, SCOPUS, CENTRAL, and CINAHL were searched for stroke randomized clinical trials using tDCS for the recovery of gait before 7 February 2022. In order to provide statistical support to the current review, we analyzed the achieved effect sizes and performed statistical comparisons.

**Results:**

A total of 24 records were finally included in our review, totaling *n* = 651 subjects. Detailed analyses revealed *n* = 4 (17%) studies with large effect sizes (≥0.8), *n* = 6 (25%) studies with medium ones (≥0.5), and *n* = 6 (25%) studies yielding low effects sizes (≤ 0.2). Statistically significant negative correlations (rho = −0.65, *p* = 0.04) and differences (*p* = 0.03) argued in favor of tDCS interventions in the sub-acute phase. Finally, significant differences (*p* = 0.03) were argued in favor of a bifocal stimulation montage (anodal M1 ipsilesional and cathodal M1 contralesional) with respect to anodal ipsilesional M1.

**Conclusion:**

Our systematic review highlights the potential of tDCS to contribute to gait recovery following stroke, although also the urgent need to improve current stimulation strategies and subject-customized interventions considering stroke severity, type or time-course, and the use of network-based multifocal stimulation approaches guided by computational biophysical modeling.

**Systematic review registration:**

PROSPERO: CRD42021256347.

## Highlights

- A systematic review of tDCS studies in post-stroke gait rehabilitation was carried out.
- Differences in clinically relevant outcomes influenced by post-stroke delay at treatment onset and stimulation strategy are reported.
- Future tDCS interventions based on analyzing activity changes are able to predict motor recovery, optimized stimulation montages, parameters, and model-based customization ought to be explored.

## Introduction

The rehabilitation of stroke sequels is among the major health problems modern societies are currently facing. Stroke stands out as the first cause of disability and the second cause of mortality in adults (1), with <20% of the affected subjects able to return to their prior professional and personal life. Although it has been classically presented as impacting old adults, stroke is becoming “*a disease of all ages”*also affecting young survivors, thereby further increasing its socioeconomic burden (2–4). Derived neurological deficits present a large heterogeneity often affecting multiple motor, sensory, and cognitive domains. The occurrence and prevalence of its symptoms are related to several variables such as the extent and location of the brain injury, subject's age, and pre-lesional neurological status (5, 6). The ability to recover close-to-normal or normal gait is one of the key symptoms in order to achieve adaptive reintegration to a functional community life. However, gait limitations are observed in more than 80% of stroke survivors (7), of which 25% will suffer from severe chronic and enduring deficits (8, 9).

Gait emerges from intrinsic excitatory-inhibitory balances within the cerebral and spinal central nervous system (CNS) and dynamic influences with the peripheral nervous system (PNS) and muscle groups. Each node, as part of a highly distributed cerebral motor network (M1, SMA, premotor cortex, cerebellum, etc.), contributes to a complex set of motor sub-processes (initiation, maintenance, error control as well as learning, planning, consolidation of human gait, etc.). Although usually compromised by the presence of hemiplegia, sensory and cognitive deficits, and walking abilities, which are much less dependent on cortical resources than fine motor skills, are often less impacted following cortical damage and cerebral-site-specific maladaptive plasticity (10). For this reason, in recent years, novel motor rehabilitation approaches (particularly non-invasive stimulation after stroke affecting the upper limbs) have attracted major interest. Unfortunately, non-motor cognitive deficits, in particular attentional and executive dysfunctions, can endure and prevent or slow down further motor rehabilitation process. For this reason, they would need to be specifically approached and facilitated (11–15).

Some well-identified functions of the healthy brain seem to be modulated through mutually inhibitory trans-callosal interhemispheric interactions able to maintain homeostatic excitatory/inhibitory balance in areas of the CNS. Roughly speaking, after a stroke, activity in the damaged hemisphere has been shown to decrease while contralateral homologous areas increase, further inhibiting the damaged hemisphere and preventing spontaneous remapping and recovery in perilesional areas sharing similar connectivity features and behavioral contributions (16). Accordingly, balance in the rivalrous transcallosal inhibitory drive has been positively associated with the degree of spared motor function and ultimately with recovery rates (17). Recovery can be achieved by directly increasing the excitability of perilesional regions in the damaged hemisphere; by suppressing homotopic spared regions of the contralesional hemisphere; the former and the latter simultaneously, or finally by activating ipsilateral spared cortical cerebral or cerebellar regions connected with peri-lesion areas. In such a scenario, non-invasive brain stimulation (NIBS) technologies can locally modulate activity in either peri-lesional or spared cortical areas of the lesioned or contralesional hemisphere, and rebalancing disbalanced networks has become a widely employed therapeutic strategy.

The framework and predictions posed by the interhemispheric mutually inhibitory interaction model, which placed the emphasis on the importance of networks linking spared and damaged areas, remain very influential when planning and assessing the coherence of neuromodulation strategies in stroke subjects. This is particularly important in domains such as spatial attention and awareness deficits (hemispatial neglect), language deficits (aphasia), and upper limb motor paralysis. Nonetheless, novel approaches based on the identification from large databases of stroke cases of anatomical of functional determinants of spontaneous motor recovery are claiming increasing attention. Very particularly, structural connectivity-based models have identified areas linked by lesion-impacted white matter connectivity most frequently involved in the spontaneous recovery of upper limb function following damage, as a function of stroke baseline severity and phase locking (18). Most importantly, cortical regions that if adequately modulated through NIBS might be likely to drive functional recovery can be integrated when selecting the suitability of a stimulation strategy for the rehabilitation of stroke sequels.

Among different NIBS technologies, transcranial electrical stimulation (tES), which integrates transcranial direct current (tDCS), alternating current (tACS), and random noise (tRNS) stimulation, stands out thanks to its low cost, safety, and has set promise of synergistic impact when combined with conventional interventions (19, 20). To date, the clinical potential of tRNS is yet to be fully established in upper limb paralysis or gait rehabilitation. Differently, the effects of tACS have been evaluated in upper limb stroke rehabilitation (21) and shown to increase the strength of functional coupling between the primary motor cortex (M1) and the cerebellum at the beta and gamma bands (22, 23) in association with the control of muscle ankle activity in treadmill training (24). Nonetheless, tDCS remains the technological approach most widely evaluated for motor rehabilitation, and for this same reason, the focus of the current systematic review on gait recovery following stroke.

Transcranial DCS is based on the application of a low-intensity direct continuous current able to induce sub-threshold shifts of polarity-dependent membrane potentials and ensuing long-term potentiation/depression-like plasticity (25–27). Recent, meta-analyses addressed the impact of tDCS combined with other interventions on balance and postural control (28), gait and speed rehabilitation (29), and improvements in walking ability (30–32), and despite promising results, outcomes at the group level remain either heterogeneous or inconclusive. Such a disparity has been argued to emerge mainly from the large variety of post-ictal clinical presentations, baseline symptom severity, the influence of associated factors such as the presence of hemineglect, sensory or proprioceptive deficits, and the wide array of choices in tDCS parameters (cortical target, current intensity and density, electrode size, location, montages, etc.). A majority of tDCS trials have been carried out using the so-called “classic” bipolar electrode scheme based on a single anode and a single cathode delivering up to 2-mA peak intensity. Nonetheless, a move toward the use of multi-site set-ups targeting networks rather than isolated regions and a subject-customization of stimulation parameters guided by biophysical computational models and simulations of current distribution is slowly emerging in the field (33–35).

To contribute to this effort and ultimately improve motor rehabilitation following stroke, here we reviewed the state-of-the-art in the field by means of a systematic search and analysis of scientific literature reporting uses of tES, and particularly tDCS, for post-stroke gait impairments. On the finally selected sample of high-quality studies, we first assessed and quantified by recalculating effect sizes of the current success of tDCS strategies on gait rehabilitation following stroke; and second, we explored the influence of tDCS treatment intensiveness, periodicity, current intensity, and density, post-stroke time at tDCS treatment onset, and stimulation strategy on effect sizes across available outcome measures. On such a basis, we discussed the suitability of current tDCS strategies defined in terms of stimulation parameters and cortical targets used in the rehabilitation of gait following stroke. We finally discussed and speculated about novel tDCS targeting strategies, which take into account baseline severity and clinical progress pre-treatment onset, stroke lesion features, and individual subject's anatomy and brain features through biophysical computational models might help profile more efficient interventions.

## Materials and methods

The current systematic review is presented according to the recommendations in the Preferred Reporting Items for Systematic Review and Meta-analyses: The PRISMA Statement (36). The protocol and systematic review were prospectively registered on June 2021 through PROSPERO: CRD42021256347.

###  Data sources and search strategy

The previous literature search focused on stroke studies that used tDCS for the recovery of functional balance and walking, was conducted on 7 February 2022 using web-based databases including MEDLINE (through PubMed), SCOPUS, Cochrane Central Register of Controlled Trials (CENTRAL), and CINAHL (through EBSCOhost). Keywords with regard to the intervention, domain, and condition included the following: (“tES” OR “transcranial electrical stimulation” OR “tDCS” OR “transcranial direct current stimulation” OR “anod^*^” OR “cathod^*^”) AND (“balance” OR “posture” OR “gait” OR “walk” OR “locomotion”) AND (“stroke” OR “cerebrovascular accident” OR “brain ischemia”). Reference lists were manually screened by two independent expert observers to be identified by common agreement relevant studies.

### Study eligibility criteria

Studies were included when the following criteria were met: (1) application of tES in human subjects with stroke of at least 18 years of age; (2) assessment of the effect of tES on the recovery of walk and/or balance including assessments of these parameters; (3) comparison of an active tES alone or combined with other rehabilitation approaches (excluding however other brain stimulation tools in combined therapeutic approaches); (4) randomized-controlled trials, including crossover and parallel designs; and (5) peer-reviewed articles published in English before 7 February 2022. Studies were excluded if the retrieved item (1) was a review study, a single case report, an editorial comment, or a meta-analysis of prior studies; (2) included stroke subjects with a prior history of other neurological or musculoskeletal diseases; (3) included healthy subjects or subjects with other neurological or musculoskeletal diseases as control groups; (4) consisted of abstracts with no associated full article published in a peer-reviewed English-speaking journal.

### Study selection and data extraction

Authors XC-T, AV-C, and MC independently screened the retrieved studies by carefully reading titles and abstracts. Once a final agreement between reviewers was achieved, the following information was extracted from each article: (1) author/s and year of publication; (2) study design, demographics, and clinical characteristics; (3) total number of enrolled participants, number of participants included/completed the study, and number of participants in intervention/control group; (4) subject's age; (5) subject's gender; (6) subject's stroke type and baseline severity; (7) subject's stroke phase; (8) intervention protocol design; (9) stimulated targets; (10) methods used to localize cortical targets; (11) stimulation current intensity; (12) electrode size and current density; (13) total session number; (14) duration; (15) periodicity; (16) associated interventions performed on the experimental or control group during stimulation regimes; (17) outcome measures used to report the results; and (18) effect sizes. Data were extracted using a structured table (refer to summary in Table 1).

**Table 1:** Summary of included data by each of the 24 retained individual studies.

**References**	**Design**	**Subjects N (included/finished; intervention group/control group; age; man/woman)**	**Stroke demographics (type/phase/baseline impairment)**	**Target areas and electrode positions (anodal vs. cathodal), tools used to allocate targets**	**tDCS protocol (intensity & periodicity, controls and associated interventions)**	**Outcome measures**	**Cohens'*d***
Andrade et al. (37)	RCT parallel, double blinded, sham controlled	I vs. F: 60/60 Ig vs. Cg: 15/15/15/15 Ag: 69 ± 3.2 M vs. W: 35/25	60I Acute-subacute Medium	Group A: A-M1 ipsilesional C-supraorbital contralesional Group B: A-M1 ipsilesional C-M1 contralesional Group C: A-supraorbital ipsilesional C-M1 contralesional 10/20 EEG system and anatomical limits	2mA, 5 × 7 saline-soaked sponge 0.05mA/cm^2^ 10 sessions, duration of each not reported 5 days a week × 2 weeks Intervention group: A-tDCS, C-tDCS, Bilateral tDCS + conventional physical therapy Control group(s): Sham tDCS + conventional physical therapy Concurrent rehabilitation 3h a week × 2 weeks during tDCS treatment	FSST OSI FES-I BBS 6mWT STS	BBs: Insufficient data 6MWT: Insufficient data
Cattagni et al. (38)	RCT cross-over, double blinded, sham controlled	I vs. F: 24/24 Ig vs. Cg: 12/12 Ag: 57 ± 13 M vs. W: 19/5	17I/7H Chronic Mild-medium	A-leg area of the ipsilesional motor cortex, laterally to Cz C-supraorbital contralesional 10/20 EEG system and anatomical limits	2mA, 5 × 7 saline-soaked sponge 0.06mA/cm^2^ 2 sessions (1 sham), 30 min each 1 a week × 2 weeks (7 days wash out) Intervention group: A-tDCS in rest Control group(s): Sham tDCS in rest No concurrent rehabilitation program nor online rehabilitation during tDCS	3D gait analysis EMG activity of the RF, GM, SOL and TA muscles BDNF genotype	Gait velocity (kinetic assessment): 0.07
Chang et al. (39)	RCT parallel, double blinded, sham controlled	I vs. F: 24/24 Ig vs. Cg: 12/12 Ag: 62.8 ± 10.4 M vs. W: 15/9	24I Acute-subacute Medium	A-TA area of the ipsilesional motor cortex C- supraorbital contralesional Hotspot detected with TMS MEP	2mA, 3 to 6 cm diameter saline-soaked sponge A 0.28 mA/cm^2^ and C 0.07mA/cm^2^ 10 sessions, 10 min each 5 a week × 2 weeks Intervention group: A-tDCS + conventional intensive physical therapy Control group(s): Sham tDCS + conventional intensive physical therapy Concurrent rehabilitation program 2.5 h daily, 6 days a week × 2 weeks + online rehabilitation during each tDCS session	MEP of the TA FMA-LE MI-LE FAC BBs	BBs: 0.3 Gait velocity: 0.2
Danzl et al. (40)	RCT parallel, double blinded, sham controlled	I vs. F: 8/8 Ig vs. Cg: 6/2 Ag: 67.8 ± 11.7 M vs. W: 4/4	5I/3H Chronic Medium-severe	A-center of leg area (cz) C-supraorbital area	2mA, 5 × 5 and 5 × 7 saline-soaked sponge A 0.08mA/cm^2^ C 0.05mA/cm^2^ 12 sessions, 20 min each 3 a week × 4 weeks Intervention group: A-tDCS + lokomat Control group(s): Sham tDCS + lokomat Concurrent rehabilitation program, 3 h a week × 4 weeks, and rehabilitation following each tDCS session	10 MWT TUG BBs FAC SIS-16	10 MWT: Insufficient data TUG: Insufficient data BBs: Insufficient data
Fusco et al. (41)	RCT parallel double blinded, sham controlled	I vs. F: 14/11 Ig vs. Cg:7/7 Ag: 58.36 ± 14.3 M vs. W: 5/5	11I Subacute Mild-severe	A-Ipsilesional shoulder C-Contralesional M1 10/20 EEG system	1.5mA, 5 × 7 saline-soaked sponge 0.04mA/cm^2^ 10 sessions, 10 min each 5 a week × 2 weeks Intervention group: C-tDCS + intense conventional physical therapy Control group(s): sham tDCS + intense conventional physical therapy Concurrent rehabilitation program, 1.5h daily, 5 days a week × 2 weeks following tDCS sessions	10MWT TUG 6mWT 9HPT Dynamometer FMA-UE CNS RMI BI FAC	10MWT: Insufficient data TUG: Insufficient data 6mWT: Insufficient data
Geroin et al. (42)	RCT parallel, double blinded, sham controlled	I vs. F: 30/30 Ig vs. Cg:10/10/10 Ag: 62.7 ± 6.4 M vs. W:23/7	30I Chronic Medium	A-Ipsilesional leg area C-Supraorbital contralesional	1.5mA, 7 × 5 saline-soaked sponge, 0.04mA/cm^2^ 10 sessions,7 min each 5 a week × 2 weeks Intervention group: tDCS + RAGT, overground walking Control group(s): sham tDCS + RAGT Concurrent rehabilitation program, 50 min daily, 5 days a week × 2 weeks + online rehabilitation during each tDCS session	6mWT 10MWT Kinetic evaluation FAC RMI Ashworth MI leg	6mWT:0.3 10MWT:0.18
Kindred et al. (43)	RCT parallel, double blinded, sham controlled	I vs. F: 21/18 Ig vs. Cg: 7/7/7 Ag: 64.8 ± 12.5 M vs. W: Not reported	Not reported Chronic Medium	Group A: HD 4 × 1 montage with the A-M1 ipsilesional Group B_ HD 4 × 1 montage with the C-M1 ipsilesional 10/20 EEG system + computational modeling for E-field prediction	2mA, 1cm diameter, A 0.63mA/cm^2^ C 0.15mA/cm^2^ 1 session, 20 min each 1 day Intervention group: A-tDCS, C-tDCS + ergometer pedaling Control group(s): Sham tDCS + ergometer pedaling No Concurrent rehabilitation program but online rehabilitation during each tDCS session	Gait kinematics Ground reaction forces over ground walking Corticomotor TA response MEP of the TA	Gait velocity (kinetic assessment): insufficient data
Klomjai et al. (44)	RCT- cross over, double blinded, sham controlled	I vs. F: 19/19 Ig vs. Cg: 19/19 Ag: 57.2 ± 2.8 M vs. W: 14/5	19I Subacute Medium	A-M1 ipsilesional C-M1 contralesional 10/20 EEG System and anatomical limits	2mA, 7 × 5 saline-soaked sponge 0.05mA/cm^2^ 2 sessions (1 sham), 20 min each 1 a week × 2 weeks (7 days wash out) Intervention group: bilateral TDCS + conventional physical therapy Control group(s): Sham tDCS + conventional physical therapy No concurrent rehabilitation program, but 1 h of rehabilitation following each tDCS session	TUG Five times STS Peak knee torque of extension	TUG: 0.4
Leon et al. (45)	RCT parallel, single blind, active control	I vs. F: 50/49 Ig vs. Cg: 9/17/23 Ag: 48 ± 11 M vs. W: 34/15	29I/21H Subacute Severe	Group A: A-Cz vertex C-right supraorbital area Group B: A-M1 ipsilesional C-Supraorbital contralesional 10/20 EEG system	2mA, 7 × 5 saline-soaked sponge 0.05mA/cm^2^ 20 sessions, 20 min each 5 days a week × 4 weeks Intervention group: A-leg tDCS, A-hand tDCS + daily RAGT and a complete rehabilitation intense program Control group(s): daily RAGT and a complete rehabilitation intense program Concurrent rehabilitation program,5h daily, 5 days a week × 4 weeks + online rehabilitation during each tDCS session	10MWT FAC	Group A : 10MWT:0.03 Group B: 10MWT:0.19
Liang et al. (46)	RCT cross-over, double blinded, sham controlled	I vs. F: 10/10 Ig vs. Cg: 10/10 Ag: 58.9 ± 9.5 M vs. W: Not reported	Not reported Chronic Medium	A-M1 ipsilesional, 1 cm anterior to the cranial vertex C-Supraorbital contralesional 10/20 EEG system and anatomical limits	2mA, 5 × 5 saline-soaked sponge 0.08mA/cm^2^ 2 sessions (1 sham), 20 min each 1 day every 2 weeks (14 days wash out) Intervention group: A-tDCS + center of gravity shift training in a force platform Control group(s): Sham tDCS + center of gravity shift training in a force platform No concurrent rehabilitation program, but online rehabilitation during each tDCS session	BBs Forward Reach Test 10MWT Bertec Advantage Computerized dynamic posturography	BBS:0.12 10MWT:0.23
Madhavan et al. (47)	RCT parallel, double blinded, sham controlled	I vs. F: 81/81 Ig vs. Cg: 20/20/21/20 Ag: 58.7 ± 9.7 M vs. W:55/26	53I/28H Chronic Medium	A-M1leg ipsilesional C-Supraorbital contralesional Hotspot detected with TMS MEP	1mA, 5 × 2.5 and 4.5 × 5.5 saline-soaked sponge, A 0.08 mA/cm^2^ C 0.04 mA/cm^2^ 12 sessions, 15 min each 3 a week × 4 weeks Intervention group: tDCS + AMT + HISTT, AMT + HIST, tDCS + HIST Control group(s): sham tDCS + HISTT Concurrent rehabilitation program, 3h a week × 4 weeks, prior to rehabilitation	10MWT CME 6mWT BBs SIS-16 TUG miniBESTest FMA-LE	10MWT: 0.39 6mWT:0.28 BBs:0.12 TUG:0
ji et al. (48)	RCT cross-over, double blinded, sham controlled	I vs. F: 30/30 Ig vs. Cg: 30/30 Ag: 62.9 ± 10.5 M vs. W: 21/9	17I/13H Subacute Medium	A-SMA, 3.5 cm anterior to Cz C-over the inion 10/20 EEG system	1mA, 5 × 5 saline-soaked sponge 0.04mA/cm^2^ 10 (5 sham) sessions, 20 min each 5 a week × 2 weeks (3 days wash out) Intervention group: A-tDCS + body weight-supported treadmill gait training Control group(s): Sham tDCS + body weight-supported treadmill gait training Concurrent rehabilitation program, 1 h daily, 5 days a week × 2 weeks + online rehabilitation during each tDCS session	10MWT TUG FMA-LE Tinetti-POMA Trunk Impairment Scale	10MWT:0.42 TUG:0.04 T-POMA:0.7
Mitsutake et al. (49)	RCT parallel, single blind, sham controlled	I vs. F: 34/31 Ig vs. Cg: 12/11/11 Ag: 72.6 ± 10.7 M vs. W: 19/15	28I/6H Subacute Medium	A-leg area of the ipsilesional cortex, lateral to Cz C- Supraorbital contralesional 10/20 EEG system	2mA, 5 × 5 saline-soaked sponge 0.05mA/cm^2^ 7 sessions, 20 min each Daily for a week Intervention group: A-tDCS + conventional rehabilitative intervention, A-tDCS + Functional electrical stimulation of the peroneal nerve and conventional rehabilitative intervention Control group(s): Functional electrical stimulation of the peroneal nerve and conventional rehabilitative intervention Concurrent rehabilitation program, 1h for 7 consecutive days + online rehabilitation during each tDCS session	10MWT Trunk acceleration	10MWT:0.4
Ojardias et al. (50)	RCT cross-over, double blinded, sham controlled	I vs. F: 20/18 Ig vs. Cg: 18/18 Ag: 57.4 ± 3.6 M vs. W: 12/6	15I/3H Chronic Medium	A-M1 ipsilesional, over the leg area C-Supraorbital contralesional Hotspot localized with TMS MEP and magnetic resonance	2mA, 5 × 5 saline-soaked sponge 0.08mA/cm^2^ 2 sessions (1 sham), 20 min each 1 day a week × 2 weeks (11 days wash out) Intervention group: A-tDCS in rest Control group(s): Sham tDCS in rest No concurrent rehabilitation program nor online rehabilitation during tDCS sessions	Wade test 6mWT Step length and symmetry Balance in center of pressure platform	6mWT: insufficient data
Park et al. (51)	RCT parallel, not reported, sham controlled	I vs. F: 24/24 Ig vs. Cg: 8/8/8 Ag: 59.4 ± 10.6 M vs. W: Not reported	16I/8H Chronic Mild-medium	A-Over Cz area of the left parietal lobe C-Right supraorbital area 10/20 EEG system	2mA, sponge size not reported 12 sessions, 15 min each 3 days a week × 4 weeks Intervention group: A-tDCS + TRT, TRT alone Control group(s): Sham tDCS + TRT Concurrent rehabilitation program, 30 min. daily, 3 days a week × 4 weeks + online rehabilitation during each tDCS session.	Kinematic evaluation of gait (velocity, stance phase, swing phase and step length)	Gait velocity (kinetic assessment): 0.05
Picelli et al. (52)	RCT parallel, double blinded, sham controlled	I vs. F: 30/30 Ig vs. Cg: 10/10/10 Ag: 62.8 ± 8.3 M vs. W: 22/8	30I Chronic Medium-severe	A-M1 ipsilesional C- Supraorbital contralesional 10/20 EEG system	2mA, 7 × 5 saline-soaked sponge 0.05mA/cm^2^ 10 sessions, 20 min each 5 days a week × 2 weeks Intervention group: A-tDCS + tsDCS +RAGT, A-tDCS + sham tsDCS + RAGT Control group(s): Sham tDCS + tsDCS + RAGT Concurrent rehabilitation program, 1h daily 5 days a week × 2 weeks + online.	6mWT FAC MI-LE AS Spatiotemporal gait parameters	6mWT:0.28
Picelli et al. (53)	RCT parallel, single blind, active control	I vs. F: 20/20 Ig vs. Cg: 10/10 Ag: 62.7 ± 10 M vs. W: 13/7	20I Chronic Medium-severe	Group A: A- Buccinator contralesional C-Cerebellum contralesional Group B: A-M1 ipsilesional C-Supraorbital contralesional 10/20 EEG system	2mA, 4cm diameter saline-soaked sponge 0.15 mA/cm^2^ 10 sessions, 20 min each rehabilitation during each tDCS session 5 days a week × 2 weeks Intervention group: c-tcDCS + tsDCS + RAGT Control group(s): A-tDCS + tsDCS +RAGT Concurrent rehabilitation program, 1 h daily, 5 days a week × 2 weeks + online rehabilitation during each tDCS session	6mWT FAC MI-LE AS Spatiotemporal gait parameters	6mWT: Insufficient data
Picelli et al. (54)	RCT parallel, single blind, active control	I vs. F: 40/39 Ig vs. Cg: 20/19 Ag: 64.6 ± 10.1 M vs. W: 23/17	40I Chronic Medium-severe	Group A: A- Buccinator contralesional C- contralesional cerebellum Group B: A-Buccinator ipsilesional C-ipsilesional cerebellum 10/20 EEG system	2mA, 4 cm diameter saline-soaked sponge 0.15 mA/cm^2^ 10 sessions, 20 min each 5 days a week × 2 weeks Intervention group: c-tcDCS ipsilesional + tsDCS + RAGT Control group(s):c-tcDCS contralesional + tsDCS + RAGT Concurrent rehabilitation program, 1 h daily, 5 days a week × 2 weeks + online rehabilitation during each tDCS session	6mWT FAC MI-LE AS Spatiotemporal gait parameters	6mWT: Insufficient data
Prathum et al. (55)	RCT parallel, double blinded, sham controlled	I vs. F: 26/24 Ig vs. Cg: 12/12 Ag: 57.75 ± 2.45 M vs. W: 15/9	24I Chronic Medium	A-M1 ipsilesional C-M1 contralesional 10/20 EEG system	2mA, 7 × 5 saline-soaked sponge 0.05mA/cm^2^ 12 sessions, 20 min each 3 days a week × 4 weeks Intervention group A-tDCS + Home-based exercise program Control group(s): Sham tDCS + Home-based exercise program Concurrent rehabilitation program, 1h daily 3 days a week × 4 weeks. tDCS sessions performed prior to rehabilitation	FMA Wolf Motor Function TUG Five times STS 6mWT Muscle strength assessment	TUG.0.4 6mWT:1.1
Saeys et al. (56)	RCT cross-over, double blinded, sham controlled	I vs. F: 31/31 Ig vs. Cg: 31/31 Ag: 63.2 ± 8.42 M vs. W: 17/14	29I/5H Subacute Medium	A-M1 ipsilesional C-M1 contralesional 10/20 EEG system	1.5mA, 7 × 5 saline-soaked sponge 0.04mA/cm^2^ 32 sessions (16 sham), 20 min each 4 days a week × 4 weeks Intervention group: A-tDCS + multidisciplinary intense physical and occupational therapy Control group(s): Sham tDCS + multidisciplinary intense physical and occupational therapy Concurrent rehabilitation program, 1h daily, 4 days a week × 4 weeks. tDCS sessions performed prior to rehabilitation	Tinetti-POMA RMI Trunk Impairment Scale	T-POMA: 0.94
Seo et al. (57)	RCT parallel, double blinded, sham controlled	I vs. F: 21/17 Ig vs. Cg: 8/9 Ag: 62 ± 8.9 M vs. W: 16/5	16I/5H Chronic Medium	A-leg area ipsilesional, lateral to the Cz position C-Supraorbital contralesional 10/20 EEG system	2mA, 7 × 5 saline-soaked sponge 0.05mA/cm^2^ 10 sessions, 20 min each 5 days a week × 2 weeks Intervention group: A-tDCS + RAGT Control group(s): Sham TDCS + RAGT Concurrent rehabilitation program, 1h daily, 5 days a week × 2 weeks. tDCS sessions performed prior to rehabilitation	FAC 10MWT 6mWT BBs FMA-LE Medical Research Council scale MEP of abductor hallucis	10MWT:1.33 6mWT:0.6 BBs:0.2
Tahtis et al. (58)	RCT parallel, double blinded, sham controlled	I vs. F: 14/14 Ig vs. Cg: 7/7 Ag: 61.8 ± 12 M vs. W:11/3	14I Subacute Medium	A-M1 ipsilesional C-M1 contralesional 10/20 EEG system	2mA, 5 × 5 saline-soaked sponge 0.08mA/cm^2^ 1session, 15 min 1 day Intervention group: bifocal tDCS in resting state Control group(s): sham tDCS in resting state No concurrent rehabilitation program nor online rehabilitation during tDCS sessions	Tinetti-POMA TUG	T-POMA:1.21 TUG:0.52
Utarapichat et al. (59)	RCT cross-over, single blind, sham controlled	I vs. F: 10/10 Ig vs. Cg: 5/5 Ag: 57.1 ± 12.2 M vs. W: 6/4	10I Chronic Medium	A-Ipsilesional leg motor area C-Supraorbital contralesional 10/20 EEG system	2mA, 3 × 3 and 5 × 5 saline-soaked sponge A 0.22mA/cm^2^ C 0.08mA/cm^2^ 2 sessions (1sham), 10 min each 2 days a week (48 h wash-out) Intervention group: A-tDCS in rest Control group(s): sham tDCS in rest No concurrent rehabilitation program nor online rehabilitation during tDCS sessions	Root mean square amplitude and median frequency of vastus medialis and TA TUG	TUG:0.04
van Asseldonk et al. (60)	RCT cross-over, double blinded, sham controlled	I vs. F: 10/10 Ig vs. Cg: 10/10 Ag: 58 ± 11 M vs. W: 4/6	8I/2H Chronic Medium	Group A: A-M1 ipsilesional C-Supraorbital contralesional Group B: A-M1 ipsilesional C-M1 contralesional Hotspot localized with TMS MEP and anatomical limits	2mA, 5 × 7 saline-soaked sponge 0.05mA/cm^2^ 3 sessions, 10 min each 1 day a week × 3 weeks (7 days wash out) Intervention group: A-tDCS, bifocal tDCS in rest Control group(s): sham tDCS in rest No concurrent rehabilitation program nor online rehabilitation during tDCS sessions	Kinematic assessment	Gait velocity (kinetic assessment): Insufficient data

A, anodal; AMT, ankle motor tracking; AS, Ashworth scale; BBs, berg balance scale; BDNF, brain derived neurotrophic factor; BI, Barthel index; C, cathodal; CME, corticomotor excitability; CNC, Canadian neurological scale; EMG, electromyography; FAC, functional ambulatory categories; FES-I, the falls efficacy scale International; FSST, four square step test; FMA, fulg Meyer assessment; FMA-LE, fulg Meyer assessment lower extremity; FMA-UE, fulg Meyer assessment upper extremity; GM, gastrocnemius medialis; HISTT, high-intensity speed-based treadmill training; I, included; Live rhb, a rehabilitation program runs in parallel with the interventions; MEP, motor-evoked potential; MI, motricity index; MI-LE, motricity index lower extremity; M1, primary motor area; OSI, overall stability index; Online rhb, tDCS is applied in the same session simultaneously with rehabilitation; RAGT, robot assisted gait training; RF, rectus femoralis; RMI, Rivermeald mobility index; SMA, supplementary motor area; SIS-16, stroke impact scale 16; SOL, soleus; STS, sit to stand test; TA, anterior tibial; TMS, transcranial magnetic stimulation; TRT, task related training; tsDCS, trans-spinal direct current stimulation; TUG, time up and go test; 6mWT, six-minute walking test; 9HPT, nine hold peg test; 10MWT, ten-meter walking test.

The acute phase was considered within the first 2 weeks after stroke, the subacute phase between the 2nd and the 24th week, and the chronic phase more than 24 weeks after the stroke. Classification of the baseline impairment (mild, medium, and severe) was based on the inclusion criteria of the studies and the comprehension of the baseline assessment results.

Beyond a conventional systematic review and meta-analysis on gait rehabilitation (28–32), we here linked the study outcomes with potential factors that may be bearing on gait rehabilitation. To this end, we focused on characterizing functional improvements of gait *velocity, endurance*, and *stability/mobility* in post-stroke subjects evaluated through at least one of the following clinical tests or kinetic assessments: the 10-meter walk test (10MWT), the six-minute walking test (6mWT), the Timed Up and Go Test (TUG), Tinetti-POMA (T-POMA), and the Berg Balance Scale (BBS). We assessed potential relationships with maximum effect sizes across available tasks, 24–72 h after post-tDCS treatment and treatment intensiveness (total number of sessions), and periodicity (number of sessions per week). We also assessed the relationship between effect sizes and tDCS current intensity and density. Moreover, we assessed the efficacy of tDCS interventions in relation to variables informing indirectly on post-stroke plasticity events such as time post-stroke event at treatment onset. Finally, after the inspection of the stimulation strategies used in the included studies, retained items were divided according to their stimulation strategy based on electrode location in order to assess potential relationships with effect sizes and stimulation strategies.

### Quality assessment of individual studies

Since our study focused on the modulation of motor functionality, the Physiotherapy Evidence Database (PEDro) was used to conduct study quality assessments. The PEDro scale has been proven valid and reliable (61) to evaluate the methodological quality of a set of eligible studies (62), based on the Delphi List criteria (63). It is based on the same items as the Cochrane Risk of Bias tool (CRBt) to assess the methodological quality of a clinical study, in which one point is given for each satisfied criterion. Since, item 1, which assesses external validity, is not used to calculate the PEDro total score, the final scores range between 0 and 10. Studies are considered to be of excellent quality (scores equal to or higher than 9/10), good quality (between 6 and 8/10), fair quality (between 4 and 5/10), and poor quality (lower or equal to 3/10).

### Statistical analysis

In order to understand the effect of tDCS to facilitate gait recovery in post-stroke subjects compared to other rehabilitation strategies (i.e., physical exercise, robot-assisted gait therapy, etc.), we have conducted a series of statistical comparisons exploring clinical outcome improvement after tDCS interventions taking into account the most relevant parameters linked to different tDCS strategies. First, effect sizes (Cohen's *d*) were re-calculated using the outcomes of the earliest post-treatment evaluation (usually for all studies within 24–72 h after completing the treatment), on the 6mWT to assess gait *endurance*, the 10MWT, and/or kinetic tests to explore gait *velocity*, and TUG and/or T-POMA and/or BBS for the evaluation of gait *stability/mobility*. This specific post-tDCS treatment evaluation time was selected to avoid any influence of the last tDCS session. Moreover, 24–72 h after completing the treatment was the milestone most often reported between the selected publications (16 of 24), hence allowing the fairest comparison across studies. Estimations of the effect size at ulterior post-tDCS follow-up points failed due to the lack of available data in a consistent number of studies (only five provided data for accurate calculations hindering comparison).

Second, Spearman's rank-order correlation coefficient was used to explore possible interactions between tES achieved recovery effects (effect size) in *endurance, velocity*, and *stability/mobility* over the studies and treatment *intensiveness, periodicity*, tDCS current *intensity*, tDCS current *density*, and *time since stroke* episode. To better understand the purpose of the effect, multiple regressions were complementarily conducted. Then, studies were grouped and categorized by their characteristics [treatment *intensiveness* (N sessions total, categorized into four groups: 1–2 sessions, 3–7 sessions, 8–12 sessions, and +12 sessions), *periodicity* (N sessions × week, categorized into six groups: 1 session every 2 weeks, 1 session × week, 2 sessions × week, 3 sessions × week, 4 sessions × week, and 5 sessions × week), tDCS current *intensity* (categorized into three groups: 1mA, 1.5mA, and 2mA), current *density* (categorized into three groups: ≤ 0.05, 0.05 ≤ 0.08, and 5)*, time since stroke episode* (N weeks post stroke, categorized into four groups: from 0 to 2 weeks, from 0 to 24 weeks, from 2 to 24 weeks, and + 24 weeks), and *stimulation strategy* (categorized into four groups: anodal ipsilesional M1 or SMA or leg area tDCS and contralateral supraorbital or prefrontal or inion return, cathodal contralesional M1 tDCS and ipsilesional supraorbital return, dual anodal, and cathodal M1 bilateral stimulation, cerebellar cathodal stimulation)]. After the categorization, data were tested with the Shapiro–Wilk test revealing a non-normal distribution. Then, the one-way Kruskal–Wallis test was used to analyze if there are any differences between groups within every characteristic regarding effect sizes. Only in case, the Kruskal–Wallis yielded statistically significant differences, and *post-hoc* analyses corrected for multiple comparisons using the Bonferroni method were performed. Statistical significance was set at *p* < 0.05. Note that for the *stability/mobility* outcome measures, which could be potentially represented by up to three clinical tests, when more than one test was available; statistical comparisons were computed on measures based on the mean value of the reported outcomes (nonetheless, notice this was the case of only two of the 24 studies, refer Table 1). Finally, significant results were represented in boxplots. Statistical analysis was performed using the Statistical Package for Social Sciences (SPSS, v.25.0, IBM, USA).

## Results

**Figure 1:**
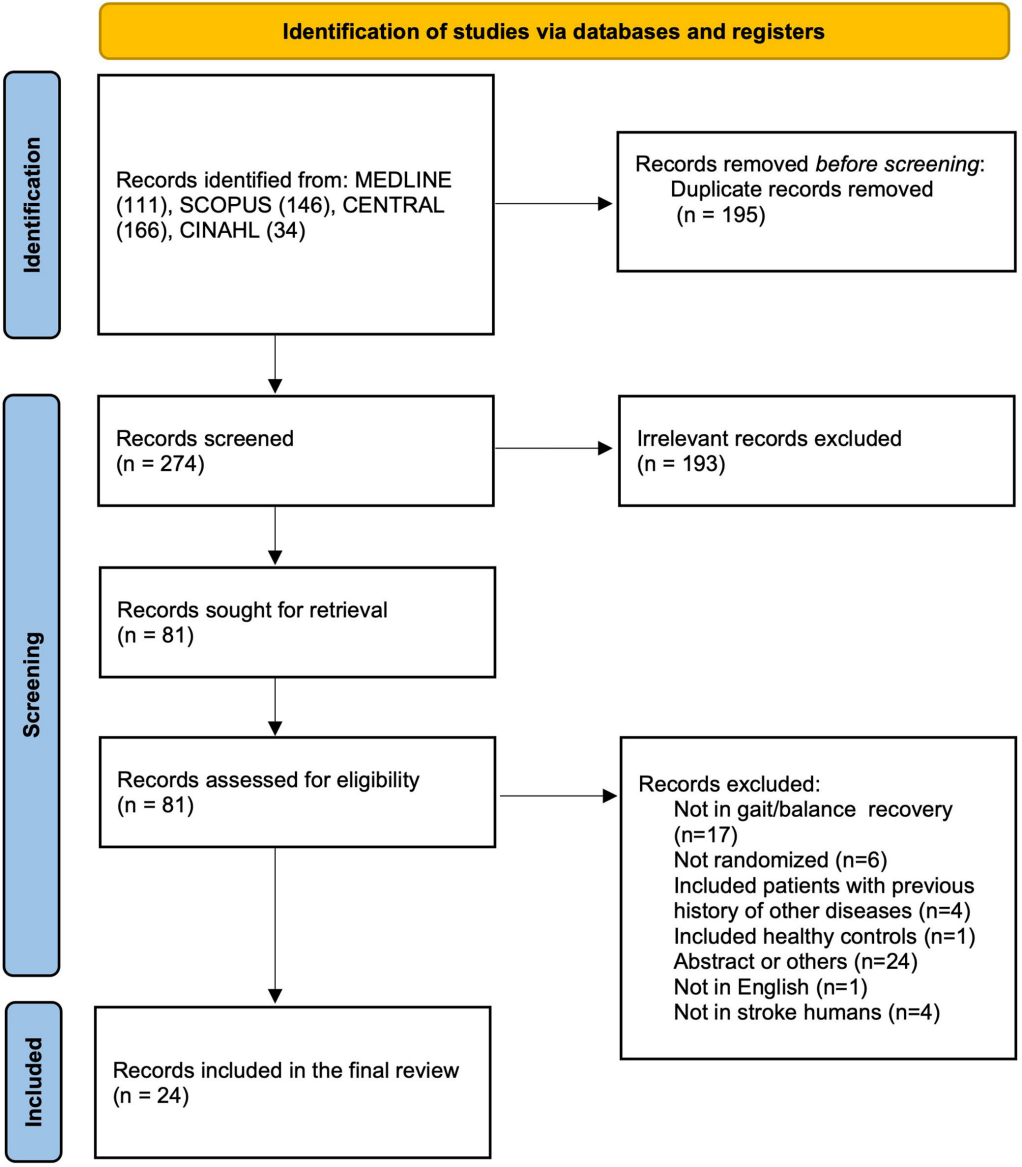
Flowchart of the literature search and study selection process.

This systematic review includes items published before 7 February 2022. After the removal of duplicates and articles that did not meet selection criteria, a total of 24 controlled randomized (of which eight crossovers and 16 parallel/five single-blinded and 19 double-blinded) trials were included, totaling 651 subjects (37–60). The PRISMA 2020 flow diagram of the search process is shown in Figure 1.

### Study selection and characteristics

A total of 457 studies were identified through the search in the four above-mentioned databases (from now on also referred to as “items”). After removing the duplicates and screening the titles and abstracts, 81 items (18%) were retained for full-text analysis by our two expert reviewers, of which 57 (70%) were excluded because they failed to meet inclusion/exclusion criteria. Of the 57 excluded items, 17 (30%) studies did not focus on the recovery of walk and/or balance neither included assessments of these parameters, six (11%) were not randomized-controlled trials, 4 (7%) included stroke subjects with a prior history of other neurological or musculoskeletal diseases, one (2%) included healthy subjects as control group lacking a sham parallel group, 24 (42%) items were not backed up by a full article published in a peer-reviewed English journal, one (2%) item was not written in English, and four (7%) worked in animal stroke models (Figure 1). All the 24 included studies were randomized-controlled trials, 16 (67%) used a parallel design, 8 (33%) a cross-over design, 19 (79%) implemented a double-blinded scheme, and 5 (21%) a single-blinded scheme; 21 (87%) had a sham control group and 3 (12%) used an active control group. The sample size ranged from 8 to 81 participants. Characteristics of the included studies are summarized in Table 1.

### Quality assessment

PEDro scores of the included 24 studies ranged from 6 to 10 and the mean quality of the studies reached a qualification of “good”; the average score for analyzed articles was 7/10. Besides, six items (25%) reached scores deemed of “excellent” quality and 18 items (33%) were considered of “good” quality. All studies specified eligibility criteria. The full PEDro assessment is shown in Table 2.

**Table 2:** Item by item PEDRO scores for each individual study included in the review.

**References**	**Item 2 randomization**	**Item 3 concealment**	**Item 4 baseline similitudes**	**Item 5 blinded subjects**	**Item 6 blinded therapists**	**Item 7 blinded assessors**	**Item 8 measures of key outcomes**	**Item 9 intention to treat**	**Item 10 reported statistical comparisons**	**Item 11 point and variability measures**	**Total/(10)**
Andrade et al. (37)	1	1	1	1	0	0	1	1	1	1	8/10
Cattagni et al. (38)	1	0	1	1	1	1	1	1	1	1	9/10
Chang et al. (39)	1	0	1	1	0	1	1	0	1	1	7/10
Danzl et al. (40)	1	0	1	1	0	1	1	0	1	0	6/10
Fusco et al. (41)	1	0	1	1	0	1	0	0	1	1	6/10
Geroin et al. (42)	1	0	1	0	0	1	1	0	1	1	6/10
Kindred et al. (43)	1	0	1	1	1	0	1	1	1	1	8/10
Klomjai et al. (44)	1	0	1	1	1	0	1	1	1	1	8/10
Leon et al. (45)	1	1	1	1	0	0	1	1	1	1	8/10
Liang et al. (46)	1	1	1	1	1	1	1	1	1	1	10/10
Madhavan et al. (47)	1	1	0	1	1	1	1	0	1	1	8/10
Manji et al. (48)	1	0	1	1	0	1	1	1	1	1	8/10
Mitsutake et al. (49)	1	0	1	1	0	1	1	1	1	1	8/10
Ojardias et al. (50)	1	0	1	1	1	0	1	1	1	1	8/10
Park et al. (51)	1	0	1	1	0	0	1	0	1	1	6/10
Picelli et al. (52)	1	1	1	1	0	1	1	1	1	1	9/10
Picelli et al. (53)	1	1	1	1	0	1	1	1	1	1	9/10
Picelli et al. (54)	1	1	1	1	0	1	1	1	1	1	9/10
Prathum et al. (55)	1	1	0	1	0	1	1	1	1	1	8/10
Saeys et al. (56)	1	1	1	1	0	1	1	1	1	1	9/10
Seo et al. (57)	1	0	1	1	1	1	0	1	1	1	8/10
Tahtis et al. (58)	1	0	1	1	0	1	1	1	1	1	8/10
Utarapichat et al. (59)	1	0	0	1	0	0	1	1	1	1	6/10
van Asseldonk et al. (60)	1	0	0	1	1	0	1	0	1	1	6/10

Items description (extracted from the official web page: https://pedro.org.au/english/resources/pedro-scale/): Item 2: subjects were randomly allocated to groups, Item 3: allocation was concealed, Item 4: groups were similar at baseline regarding the most important prognostic indicators, Item 5: blinding of all subjects, Item 6: blinding of all therapists who administered the therapy, Item 7: blinding of all assessors who measured at least one key outcome, Item 8: measures of at least one key outcome were obtained from more than 85% of the subjects initially allocated to groups, Item 9: all subjects for whom outcome measures were available received the treatment or control condition as allocated or, where this was not the case, data for at least one key outcome was analyzed by “intention to treat”, Item 10: the results of between-group statistical comparisons are reported for at least one key outcome, and Item 11: the study provides both point measures and measures of variability for at least one key outcome.

### Subjects: Demographic and individual considerations

Taken together, all studies included a total of 651 subjects of both genders (383 men and 213 women), gender remained unclear or unreported for 55 subjects. The mean average age was 60 years; 491 subjects had an ischemic stroke; 129 subjects were reported as having suffered a hemorrhagic stroke, whereas stroke type remained unspecified for 31 subjects. It should be noted that for most of the participants, the stroke episode was the first-ever ischemic event. Regarding time post-stroke, nine studies (37%) included subjects recruited in the acute-subacute phase and 15 (62%) at the chronic stage. The 24 retained studies were classified into three different levels (mild, medium, and severe) according to the severity of post-stroke impairment at baseline of their cases and their pre-defined severity inclusion criteria. In that context, two studies (8%) included subjects with a mild-medium baseline impairment, 14 (58%) subjects with a medium baseline impairment, 6 (25%) subjects with a medium-severe baseline impairment, whereas 1 (4%) and 2 (8%) studies included, respectively, only severe or mild-to-severe subject cases.

### TDCS methods

All 24 studies (100%) aimed to manipulate a single cortical target either excitatorily or inhibitorily, through the application of tDCS using saline-soaked sponges of different sizes. The vast majority of the selected studies, 23 out of 24 (96%), used bipolar montages (i.e., one anode and one cathode), with the exception of a single study (4%) (43), that used a high-density stimulation montage (4 × 1 HD-tDCS system). In the same vein, the vast majority of reports, 22 out of 24 (91%), based their interventions on upregulation and/or downregulation of motor cortical areas through the modulation of trans-callosal rivalrous interactions. Current intensities and current densities varied from 1 to 2 mA and from 0.04 mA/cm^2^ to 0.63 mA/cm^2^, respectively. Note that, in our review, a low number of tDCS sessions were not considered an exclusion criterion. Accordingly, we distinguished two types of study designs: those that sought a therapeutic “cumulative effect” by applying several consecutive tDCS sessions (equal or more than five sessions) and those whose design was “experimental” and delivered a few sessions (less than five sessions). The total number of delivered tDCS sessions per treatment among all included studies oscillated between 1 and up to 20 active tDCS sessions. However, from those trials seeking a cumulative therapeutic effect (16 out of 24, i.e., 66%), the large majority of them (15 out of 16, i.e., 93%) delivered ~10 sessions with a periodicity of three to five sessions per week and an average duration of 10–20 min of tDCS per session. The vast majority of selected studies were carried out on chronic stroke subjects (15 out of 24, 62%) and focused on the modulation of ipsilesional M1 (21 out of 24, 87%) resources *via* an indirect effect on spared contralesional M1 through trans-callosal inhibition. A single study (4%) investigated the effects of tDCS on the supplementary motor area (48), whereas two studies (8%) used the cerebellum as a stimulation target (53, 54). Electrode allocation was based on the 10/20 EEG system in 14 of 24 studies (58%), based on the 10/20 EEg system considering anatomical limits in 4 of 24 (16%), based on hotspot localization with TMS MEP in 4 of 24 (16%) and not reported in two studies (8%). None of them used neither MRI-based neuronavigation systems nor E-field individualized predictions. Finally, from all the selected 24 studies, only one (4%) reported having used a standard healthy MRI-based finite element (FEM) biophysical computational modeling to predict electrical current distribution and optimized *ad hoc* electrode montage accordingly (43).

### Outcome measures and statistics

The current review focuses on improvements of function captured by well-established clinical tests related to gait *velocity, stability/mobility*, and *endurance* sub-functions, all contributing to gait ability which can be measured and monitored separately but is poorly informative when considered in isolation. Statistical outcomes should be interpreted cautiously given the differences between studies regarding the number of sessions, subject clinical profile, and baseline severity or the quality and availability of data. Additionally, some of the 24 studies retained for this review may have used different and non-purely clinical assessment measures than those our systematic focused on to assess tDCS impact on gait recovery which is the topic at the core of the current paper (such TMS evoked motor evoked potentials (MEP) or spatio-temporal gait parameters extracted from kinematic or electromyographic analyses). Hence, it might be the case that effect sizes calculated with such non-direct clinical measures, which we will not report on, are generally larger due to their higher sensibility in spite of being less informative for the clinically relevant gait improvements.

If one classifies the 24 selected studies considering the maximum and grand average of effect sizes across all reported tests assessing the impact of tDCS on gait and balance (between 24 and 72 h post-treatment offset) included in our selection of tasks and independently of their individual statistical significance (refer Table 1), four studies (16%) reported large effect sizes (≥0.8), six reports (25%) medium effects sizes (≥0.5), whereas, in six studies (25%), stimulation yielded low effects sizes (≤0.2). Note that *velocity* outcome measures (allowing effect size recalculation) were reported in 11 of the 24 included studies, *stability/mobility* outcomes in 10 of 24 studies, and *endurance* outcomes in only four of 24 studies. Importantly, essential data necessary to estimate effect sizes were missing for eight studies (33%) and hence could not be added to these analyses.

The four studies in which tDCS yielded large (across all different reported tasks) effect sizes involved subacute and chronic subjects who experienced significant improvement effects on mobility, gait velocity, and/or endurance, after applying either bifocal tDCS (anodal M1 ipsilesional, cathodal M1 contralesional) or anodal stimulation to the ipsilesional M1 with a cathodal supraorbital contralateral return (55–58). The number of active sessions applied in these four reports varied from 1 to 12 sessions, with intensities from 1 to 2 mA and current densities between 0.05 and 0.08 mA/cm^2^. Importantly, nearly all of these tDCS studies (three out of four) carried “live” rehabilitation programs for gait and/or balance training (in regimes of multiple days), and undertook tDCS before the onset of rehabilitation sessions, hence avoiding “online” rehabilitation (i.e., performed in temporal simultaneity with tDCS treatment). Additionally, it should be noted that the pre-therapeutic “*proof of principle*” study by Tahtis and colleagues (58) even if classified among those showing high effect sizes, consisted of a single tDCS session delivered at rest without associated rehabilitation, and hence cannot be directly compared to the remaining three studies, which delivered a regime of several days of stimulation in search of lasting effects.

The six studies yielding medium effect sizes delivered mainly anodal tDCS on the ipsilesional M1 positioning the return electrode on the supraorbital contralateral area or cathodal stimulation on the contralesional spared M1 positioning the anode in the prefrontal ipsilesional cortex (39, 42, 44, 47–49). The number of active-delivered daily sessions varied from 1 to 20 at intensities from 1 to 2 mA and current densities between 0.04 and 0.08 mA/cm^2^. Importantly, four of the six studies were coupled to an online (hence simultaneous) motor rehabilitation program involving balance and/or gait training during tDCS stimulation, one study implemented a rehabilitation program immediately following each tDCS intervention, whereas one study did not couple stimulation to a planned rehabilitation program reports that tDCS sessions were followed by 1 h of motor activity.

Finally, the six studies reporting low effects applied anodal tDCS over the ipsilesional M1 leg motor area or on a scalp position lateral to Cz (according to EEG 10/20 reference system) with the cathode on a supraorbital contralateral site. The number of active sessions varied from 1 to 20 tDCS active sessions at intensities of 2 mA in all cases and current densities between 0.05 and 0.22 mA/cm^2^. In three of these six studies, stimulation was combined with concomitant rehabilitation during participation in the protocol and also online rehabilitation during tDCS delivery (45, 51, 52); in one of them, online rehabilitation was combined during tDCS sessions in the absence of any associated concurrent rehabilitation program during the participation in the protocol (46). Finally, two studies applied tDCS without online concurrent rehabilitation during tDCS or any other type of rehabilitation (38, 59).

Regarding the eight studies for which we were unable to calculate effect sizes due to insufficient data in any of the outcome measures, five of them reported statistically significant improvements following active tDCS compared to sham stimulation (37, 40, 50, 53, 60); whereas, in contrast, three reports failed to find statistically significant effects of stimulation (41, 43, 54). The number of active tDCS sessions varied from 1 to 12 sessions using intensities ranging from 1.5 to 2 mA and current densities ranging between 0.05 and 0.63 mA/cm^2^. The tDCS strategies used by such eight studies were either anodal stimulation over the ipsilesional M1, cathodal stimulation over the contralesional spared M1, or bifocal bi-hemispheric tDCS. For three of the eight studies, stimulation sessions were combined with online rehabilitation and while participants also followed concurrent rehabilitation programs during their participation, in one study, tDCS was immediately followed by a rehabilitation session, and one study applied tDCS along online rehabilitation but in the absence of any concurrent rehabilitation program, whereas two studies simply applied tDCS at rest without any kind of either ongoing or concurrent rehabilitation.

Finally, regarding our statistical analysis, Spearman's correlation revealed significant negative correlations between the effect size of tDCS impact on *stability/mobility* measures and the number of elapsed weeks post-stroke at treatment onset (rho = −0.65, *p* = 0.04). Additionally, a one-way Kruskal–Wallis test revealed statistically significant differences (*p* = 0.04) between these same outcomes at the group level. *Post-hoc* analysis revealed differences in treatments onsetting at the subacute stage (2–24 weeks) compared to the chronic stage (+24 weeks) as a group analysis (*p* = 0.03). These outcomes strongly argue in favor of an early (*subacute*) compared to a late (*chronic*) clinical therapeutic window of opportunity, with a higher potential for effective tDCS neuromodulation. Even so, given that independent variables can together affect dependent variables, we conducted standard multiple regression to better understand our results taking *stability/mobility* measures as dependent variables and treatment *intensiveness, periodicity*, tDCS current *intensity*, tDCS current *density*, and *time since stroke* as independent variables. However, no significant results were reached (*F*_(5, 4)_ = 0.69, *p* = 0.65, *R*^2^ = 0.46) and any of the independent variables proved to be significant predictor of *stability/mobility* improvement (*p* = 0.73, *p* = 0.49, *p* = 0.87, *p* = 0.53, *p* = 0.24, respectively).

**Figure 2:**
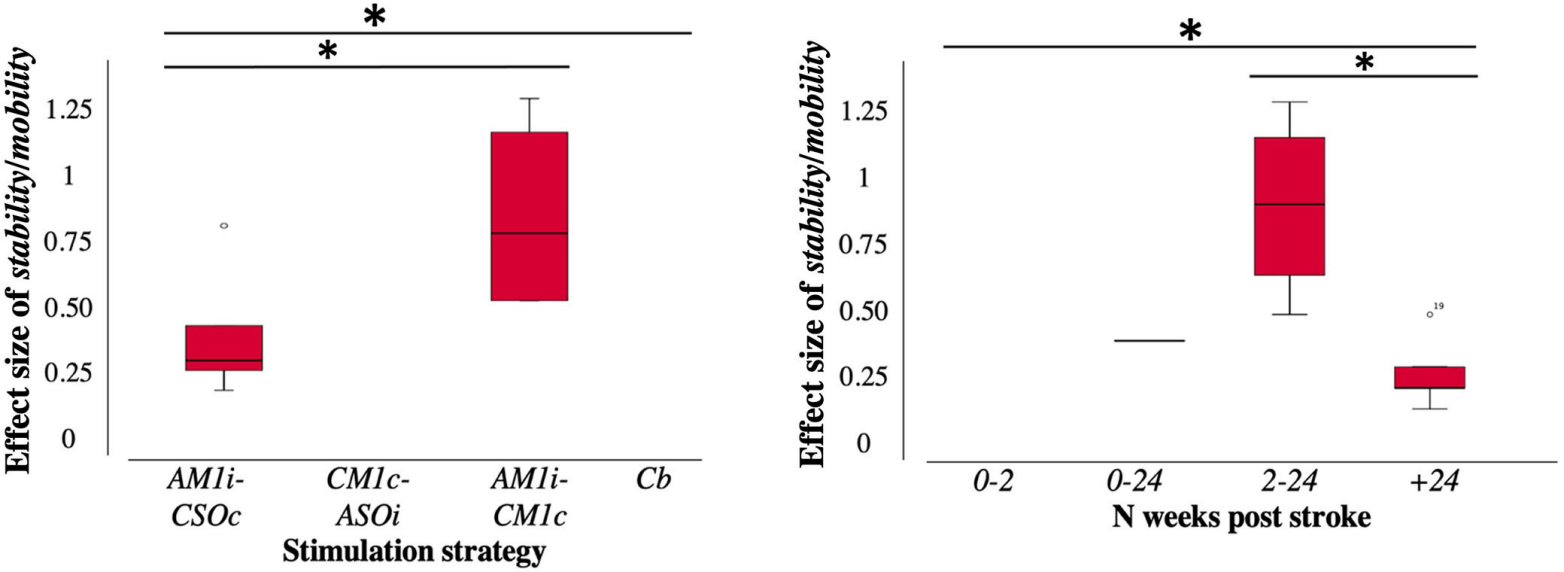
Distribution of effect sizes according to the characteristics of the retained studies (*n* = 24 publications included). Study characteristics were grouped and categorized [time since stroke episode (*N weeks post-stroke*, categorized in 4 groups: from 0 to 2 weeks, from 0 to 24 weeks, from 2 to 24 weeks, + 24 weeks) and *stimulation strategy* (categorized in 4 groups: anodal ipsilesional M1 or SMA or leg area tDCS and contralateral supraorbital or prefrontal or inion return, cathodal contralesional M1 tDCS and ipsilesional supraorbital return, dual anodal and cathodal M1 bilateral stimulation, cerebellar cathodal stimulation)]. Only measures that showed statistically significant differences in the Kruskal Wallis test are presented in the figure form stability/mobility effect size with respect to time since stroke (N weeks post-stroke) and stimulation strategy (electrode positions). Boxplots were drawn from the 25th to the 75th percentile and the horizontal line indicates the mean. Boxplots were generated in SPSS (IBM, USA). Effect size representation in boxplots was normalized from 0 = minimum, to 1.3 = maximum. AM1i, anodal primary motor cortex ipsilesional; ASOi, anodal supraorbital ipsilesional; Cb, cerebellum; CM1c, cathodal primary motor cortex contralesional; CSOc, cathodal supraorbital contralesional. *Statistically significant, *p* < 0.05.

On the other hand, a Kruskal–Wallis test reached significance (*p* = 0.03) between the effect size of tDCS on *stability/mobility* and stimulation strategy arguing in favor of the bifocal stimulation montage (anodal M1 ipsilesional and cathodal M1 contralesional) with respect to anodal M1 ipsilesional stimulation alone with supraorbital contralesional cathodal return. No other statistically significant correlations or group differences were found between effect sizes in *velocity* or *endurance* vs. treatment *intensiveness, periodicity*, tDCS current *intensity, stimulation strategy*, tDCS current *density*, and *time since stroke* episode, or between effect sizes in *stability/mobility* vs. treatment *intensiveness, periodicity*, tDCS current *intensity*, or tDCS current *density*. Data distribution of the two measures showing significant group differences is represented in Figure 2.

## Discussion

The current systematic review summarizes and evaluates peer-reviewed studies published in English language journals until 7 February 2022, assessing the use of tDCS for gait rehabilitation in stroke subjects. We aimed to characterize current state-of-the-art and assess the efficacy of most common strategies and methodologies to improve future therapeutic applications in stroke subjects for gait and associated motor and cognitive deficits. Our systematic review also implemented a complementary statistical strategy to explore the influence of time post-stroke at treatment onset, tDCS current intensity and density, tDCS treatment intensiveness, tDCS treatment periodicity, and tDCS strategy on effect sizes achieved by individual studies on tasks assessing gait *velocity, endurance*, and *stability*/*mobility*. The influence of these specific parameters was explored to shed further light on how they might impact modulatory ability so that the most efficient stimulation parameters and strategies could be planned to maximize post-injury plastic adaptive reorganization across specific windows of opportunity and subject's conditions.

Our review work identified at least 10 studies (out of 16 items with available data, and a total set of 24) with moderate or high effect sizes suggesting that tDCS remains a promising strategy to facilitate the recovery of different aspects of gait following stroke. Nonetheless, according to prior meta-analyses on this topic, our own analyses on general effect sizes revealed a rather modest impact at the group level (28–32). On such a basis, we emphasize the role of inter-individual differences in response to treatment (in turn determined by factors such as baseline clinical severity and time between stroke event and treatment onset or stimulation strategy and interactions thereof) as responsible for weakening therapeutic impact in large subject cohorts. In this context, we hypothesize that therapeutic individualization might be one of the key strategies to improve the clinical success of tDCS on gait rehabilitation following stroke. This effort first needs to be articulated by designing clinical trials which stratify subjects on the basis of baseline symptom severity at the time of treatment onset and “tailored” neuromodulation strategies based on pre-defined biomarkers predictive of beneficial responses to tDCS treatment such as (among additional potential others): corticospinal tract integrity (64–68), lesion location, extent, time post-injury and state of ongoing activity on lesional, perilesional, and spared associated areas (18, 69–73). Unfortunately, to date, very few of these relevant variables have been systematically tested in clinical trials and their influence had been explored in multivariate studies combining them rather than addressing each one at a time.

### Neurostimulation strategies for gait rehabilitation

**Figure 3:**
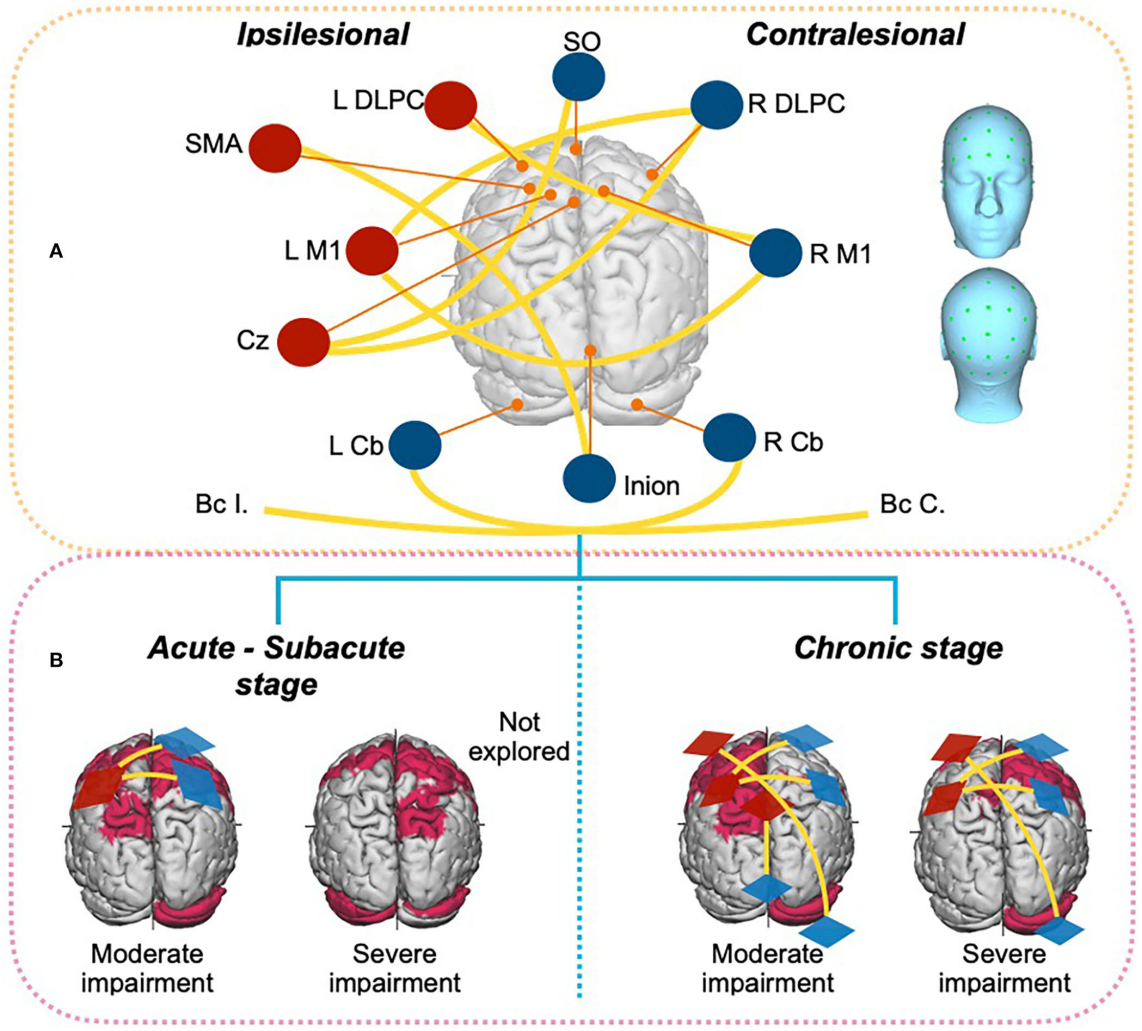
Schematic drawing presenting the types of tDCS targets and stimulation strategies for anode and cathode placing used for gait rehabilitation following a motor stroke (data is summarized and presented in the example for a left hemisphere stroke). **(A)** Different tDCS montages used by the studies (*n* = 24 publications) retained in the current review, presented on a top view of a brain in relation to the targeted region (in labels). Anodes are indicated in red whereas cathodes are shown in blue. Yellow lines represent the anode-cathode connection of each montage. **(B)** Most used tDCS montages at the subacute or acute stage (left) or the chronic stage (right) following stroke considering symptom baseline severity (moderate vs. severe impairment) extracted from the studies (*n* = 24) retained for the current review paper. Red shadowed brain areas signal sites for induced tDCS activity likely beneficial to motor recovery [according to Koch et al. (18)]. BcC, Buccinator contralesional; BcI, buccinator ipsilesional; C, Contralesional; Cb, Cerebellum; Cz, Cz position in 10/20 EEG system; DLPC, Dorsolateral Prefrontal cortex; I, Ipsilesional; L, left; M1, Primary motor; R, Right; SM, Sensorimotor cortex; SMA, Supplementary motor area; SO, Supraorbital.

Our detailed inspection of available evidence for the effects of tDCS on gait rehabilitation following stroke according to well-stated criteria (a total of 24 publications included in the analyses of the current review) demonstrates that (as is the case for upper-hand rehabilitation peer-reviewed research) a large majority of therapeutic tDCS stroke studies in this field rely on the manipulation of trans-callosal interhemispheric inhibitory interactions (17, 74) (Figure 3A). Furthermore, our own statistical analyses suggest that immediate post-treatment effect sizes across individual studies and outcome *stability/mobility* measures scaled significantly (rho = −0.65, *p* = 0.04) with time post-stroke, and show that the implementation of tDCS rehabilitation programs during the subacute phase achieved higher effect sizes than those acting at later stages. More specifically, interventions onsetting up to 2–24 weeks post-stroke (sub-acute period), when perilesional and spared brain systems are more prone to functional reorganization, may have more chances to achieve clinical success than those operating at a later chronic stage.

The current systematic review and associated analyses also suggest that tDCS might be more prone to increase the activity of spared residual perilesional networks around the areas of stroke damage, hence better suited than the manipulation of spared/intact remote circuitry (with similar input-output connectivity patterns and neurophysiological coding strategies for the impaired functions) to effectively support the remapping of lost functions. Concomitantly, contralesional hyperactivity generated by a partial loss of the inhibitory drive from the injured hemisphere may play a compensatory role, acting as a recovery-promoting mechanism, especially at acute stages following large lesions (18, 75). In this regard, the inhibition of perilesional activity mediated by a local over-expression of extra synaptic GABA_A_ receptors in neuronal resources hosted in the penumbra periolesional region around the stroke area and beyond has been reported as persisting for more than a month. Most importantly, reversing this inhibition has been shown to improve motor prognosis (76, 77). The latter observation supports the notion that the modulation of such activity from either local (i.e., by enhancing perilesional activity directly on the stroke region) or distant (i.e., by suppressing areas from the spared contralateral hemisphere exerting an abnormally intense inhibitory drive onto perilesional regions) targets is key to achieving some level of functional remapping and benefit clinical recovery.

Similar “*push and pull*” rivalrous interaction mechanisms mediated by mutually inhibitory projections have also been reported between nodes of widespread cerebral and cerebellar motor networks. In this regard, during the transition from the acute to the subacute post-stroke phase, decreases in ipsilesional M1 function contrasting with lesion size and location-dependent activity increases of ipsilesional premotor regions, supplementary motor area (SMA), the contralesional cerebellum, and the contralesional M1 have been reported and interpreted as a spontaneous brain compensatory reactions able to preserve motor output (75, 78, 79). On such basis, most studies focusing on post-stroke gait rehabilitation in the sub-acute phase have opted for strategies inhibiting contralesional M1 activity and/or by exciting ipsilesional M1 systems, yielding significant improvements at the group level. Nonetheless, such effects have been proven to be quite heterogeneous across subjects and studies. Moreover, a lack of clinically relevant effects has also been often reported for net excitatory effects of stimulation delivered to the ipsilesional hemisphere.

In such context, the notion of a bell's shape function relating post-stroke clinical severity at baseline and levels of functional recovery, emphasizing the importance of a specific window of gait impairment that maximizes a beneficial impact of stimulation has emerged in the domain. Exactly to this regard, recent connectivity-based clinical predictive models exploring the influence of damaged white matter projections in stroke subjects (18), based on fractional anisotropy measures, suggest that subjects with mild-to-intermediate baseline impairments showing greater levels of sparing in relevant bilateral structural connectivity bundles [involving not only in primary motor (M1), ventral premotor (PMv) but also the ipsilesional inferior frontal gyrus (IFG), and bilateral parietal cortices, somatosensory areas and attentional regions] tended to achieve greater recovery levels than those in which such connectivity was damaged (Figure 3B.1). Nonetheless, 3-month post-injury, relevant structural connectivity of moderately injured subjects associated with significant spontaneous recovery changes, particularly when subcortical structures of the injured hemisphere were affected, encompassed the contralesional cerebellum, and of frontal, premotor, and somatosensory areas. Three months after stroke, spontaneous plasticity processes have been shown to weaken, and at such an advanced stage, sustained contralesional hyperactivity is associated with worse clinical outcomes, hence considered deleterious for the recovery of mild motor impairment, whereas paradoxically, it might remain beneficial for large corticospinal tract lesions (73, 78–80). This same predictive model reveals that for subjects with mild-to-intermediate motor impairments, relevant structural connectivity warranting recovery varies with respect to severely affected subjects, highlighting the need for individualized interventions (18).

All in all, severity- and phase-dependent vicariation (81) and neural compensation by functional remapping or reorganization in novel pathways and cortical and subcortical areas (including the cerebellum) underline the chances for adaptive plasticity and clinical recovery of motor function (75) (Figure 3B.2). Most importantly, the dynamic nature of such phenomenon can be captured by computational models, which might anticipate prognosis and inspire more effective subject-customized therapies to regain motor abilities. However, current connectivity-based predictive models for motor recovery have been developed for upper limb impairments, hence set up rules might not necessarily directly apply and adequately inform gait rehabilitation, highlighting the need to extend such work (18) to different motor, sensory, and cognitive domains.

### Identifying optimal strategies to increase TDCS efficacy in post-stroke gait rehabilitation

Our analyses revealed statistically significant correlations (*p* = 0.03) between the effect size of tDCS treatment on *stability/mobility* and stimulation strategy, arguing in favor of the bifocal stimulation montage (anodal M1 ipsilesional and cathodal M1 contralesional) with respect to anodal M1 ipsilesional stimulation alone with supraorbital contralesional cathodal return. This suggests that tDCS studies implementing strategies coherent with current knowledge or state-of-the-art connectivity-based predictive models, sensitive to stroke phase-specific activity changes ultimately tied to spontaneous adaptive plasticity may have the potential to result in greater clinical effects than those that did not. This outcome highlights the importance of closely monitoring and characterizing (from the acute to the chronic post-stroke phase) brain activity dynamics related to adaptive spontaneous plasticity experienced by subjects and using stimulation to guide and promote that in order to optimally enhance recovery.

Additionally, technical aspects related to tDCS delivery such as the electrode placement, the electrode montages, or the magnitude of delivered currents but most importantly predicted current density (V/m) on the pursued cortical target have been found to influence therapeutic outputs. Whereas, in contrast, no significant correlations were found between post-tDCS effect sizes and delivered current intensity (in mA) or density (intensity/electrode surface). Further attempts to identify potential relationships between the effect sizes of tDCS impact and the latter factor failed due to the lack of available data to accurately estimate such parameters in a significant number of studies. Nonetheless, one main and single reason explains this outcome; delivered field intensity (in mA) which was indeed well reported in a majority of studies or electrode current density (in mA/cm^2^) inform poorly on peak current density (V/m) achieved at a given cortical target. In the absence of direct intracranial recordings, an accurate estimation of electric field strength (|*E*|) or the normal component of the electric field (nE) at target would require the computation of a head and brain biophysical model simulating current distribution based on individual subject MRI-based models (at worst, on a standard representative MRI volume) which a great majority of studies lacked (34, 82, 83). To overcome this limitation in the future, studies should be encouraged to include model-based estimations of the predicted electrical field (specially |*E*| and |nE|) revealed by standard or individualized biophysical head–brain FEM models.

Most current tDCS multichannel systems (i.e., using more than two electrodes) allow for the simultaneous stimulation of different cortical areas. Moreover, the uses of such technologies guided by MRI-based computational biophysical modeling systems such as NeMo-TMS (84) ROAST (85), or SimNIBS (86) can contribute to a more precise control of the distribution of electrical flow, providing a tool to fulfill subject-customized optimized stimulation strategies. Unreasonably, despite biophysical FEM computational models are being used in other clinical settings is yet to become mainstream to be used in post-stroke motor rehabilitation, to adequately dose tDCS and assist the design of individually tailored interventions. Regardless, precise and reliable targeting informed by current distribution models taking into account individual anatomical head/brain models is not the only variable that must be mastered to adequately predict outcomes. For example, given that stroke is indeed a network dysfunction, the status of time-correlated oscillatory activity and interregional synchrony operating at different frequency bands seems also paramount in this regard. More specifically, decreases in posterior alpha (10–12 Hz) and beta-band oscillation power (14–20 Hz, closer to the lesion), global increases of delta (1–4 Hz) and theta (4–8 Hz) synchronization, broadband interhemispheric oscillatory asymmetries (15–50 Hz) with lower power in the injured hemisphere and alpha and beta power decreases related to changes of functional connectivity (87–89) are some post-stroke electrophysiological features considered for optimizing therapeutical interventions at the group or the individual level. Once such individual biomarkers are tested and validated, two tES modalities, Transcranial Alternate Current Stimulation (tACS) and Random Noise Stimulation (tRNS), are able to entrain and desynchronize, respectively, beta- and gamma-related activity in motor networks, open the possibility to design novel stimulation strategies which might show higher efficacy by manipulating the synchrony and correcting the abnormal rhythms, rather than simply operating on the activity levels.

### Setting the stage for future innovative TDCS strategies in the rehabilitation of stroke

**Figure 4:**
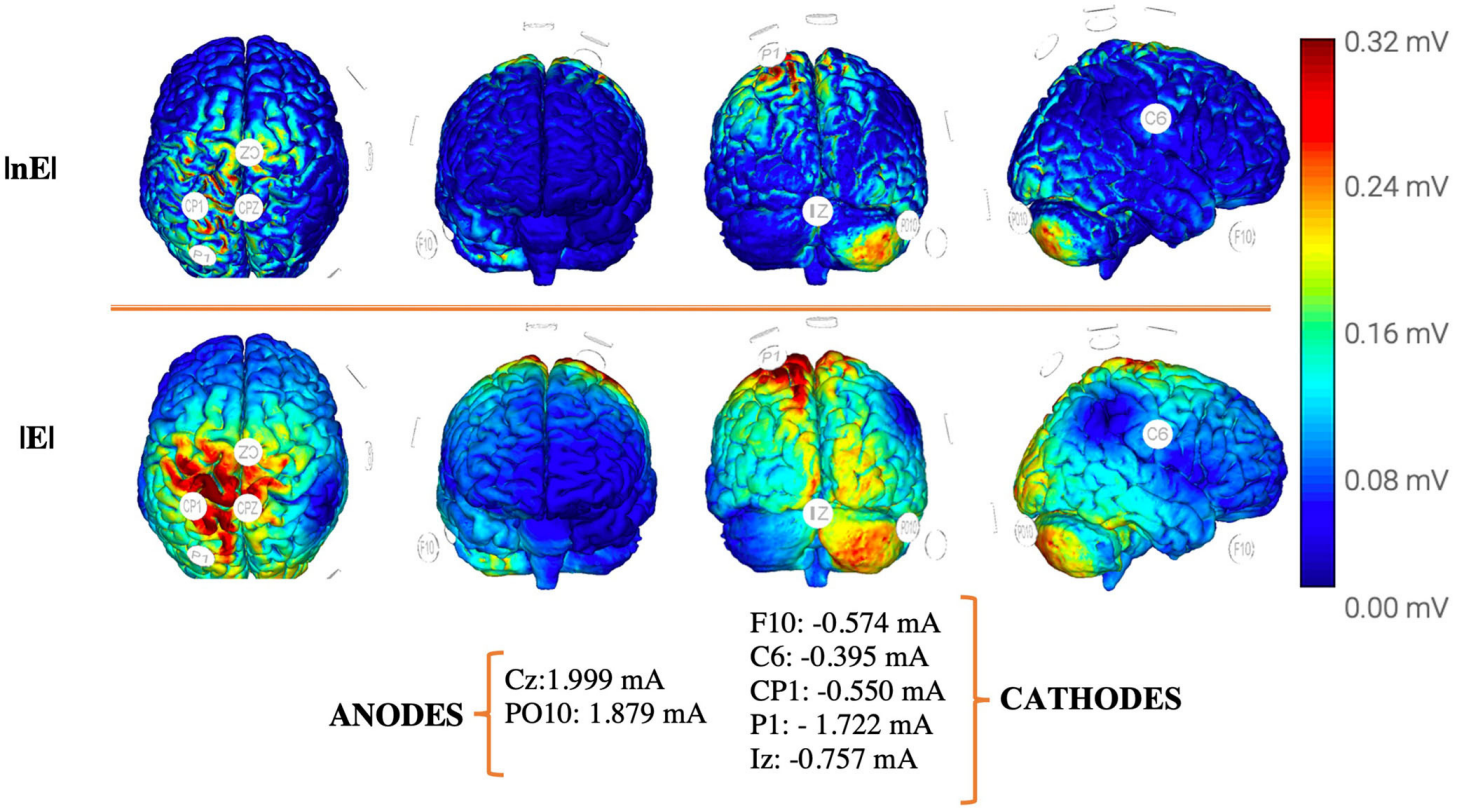
Proposal for an optimized multipolar stimulation solution for ‘gait/motor rehabilitation' [see Otal et al. (90)] following a unilateral stroke motor lesion affecting the left hemisphere, based on a standard 8 channels tDCS device, electrode size: πcm^2^ Ag/AgCl, aiming at delivering 0.25 V/m at each target [(1) M1 ipsilesional, (2) SM cortex ipsilesional, and (3) anterior cerebellum contralesional] on the basis of 4mA total injected current, and a constrained by a maximum of 2 mA on each individual electrode. Model optimization has been generated from a *headreco* routine (FEM) of the MNI152 template in SimNIBS 3.2.3 maximizing focality while controlling the E-field strength. Pictures were extracted from the Neuroelectrics Instrument Controller® interface. Cb, cerebellum; DLPC, dorsolateral prefrontal cortex; M1, primary motor; SM, sensorimotor cortex; V/m, volts per meter; E, total field strength; nE, normal component of the electric field.

Promising tDCS stimulation strategies and predictive models based on spurring beneficial (i.e., adaptive) dynamic changes or limiting maladaptive reorganization following stroke for rehabilitation are continuously being proposed. Given current knowledge on the importance of involved structural connectivity for motor rehabilitation depending on lesional stage and damage severity, plus the added possibility of using multichannel (hence multipolar or multi-site) tDCS systems combined with new electric flow distribution prediction algorithms with higher focality, two innovative approaches that to date have never been systematically developed are called to gain momentum; however, our propositions still need to be clearly discussed and explored in controlled trials to assess their feasibility. First, as proposed by Otal et al., a multi-site stimulation strategy aiming to boost the re-learning and consolidation of motor skills could be achieved by driving simultaneous activity increases of ipsilesional sensorimotor cortex S1, ipsilesional M1, and the anterior lobe of the contralesional cerebellum, while simultaneously decreasing contralesional M1 contributions (Figure 4). In the same vein, a viable alternative adapted to even more severe subjects may consist of upregulating the activity of the ipsilesional M1 while downregulating activity in contralesional primary sensorimotor areas S1, contralesional M1, and contralesional anterior cerebellum (90).

**Figure 5:**
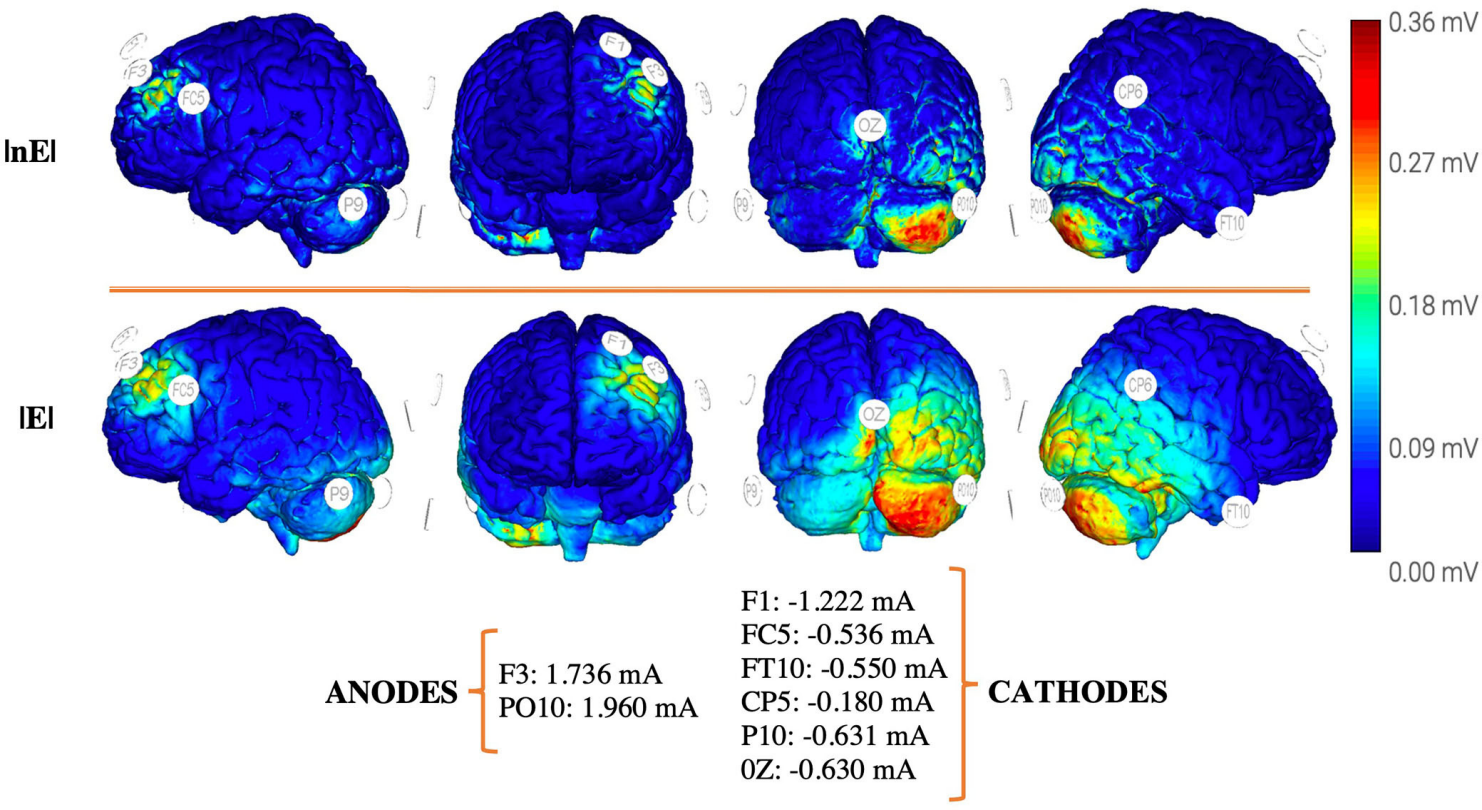
Proposal for an optimized multipolar stimulation strategy implementing “combined cognitive/motor rehabilitation” [see Wessel and Hummel (91)] following a unilateral stroke motor lesion in the example affecting the left hemisphere, based on a standard 8 channels tDCS device, electrode size: πcm^2^ Ag/AgCl, aiming at delivering 0.25V/m at each target [(1)DLPC ipsilesional and (2) anterior cerebellum contralesional] on the basis of 4mA total injected current, and a constrained by a maximum of 2 mA on each individual electrode. Model optimization has been generated from a *headreco* routine (FEM) of the MNI152 template in SimNIBS 3.2.3 maximizing focality while controlling the E-field strength. Pictures were extracted from the Neuroelectrics Instrument Controller® interface. Cb, cerebellum; DLPC, Dorsolateral Prefrontal cortex; V/m, volts per meter; E, total field strength; nE, normal component of the electric field.

Second, some studies have recently shown that motor and attentional systems [particularly the dorsal attentional network (DAN)] interact during stroke rehabilitation and influence chances for recovery (6, 13, 14). On such basis, a multi-site and multi-domain approach (15, 91) aiming to simultaneously increase activity in the dorso-lateral prefrontal cortex (DLPC) and the cerebellum in moderate-to-severely affected subjects in subacute or chronic stroke stages (refer Figure 5) could potentiate the therapeutic effect of tDCS on post-stroke gait impairments by acting in a synergistic manner on two separate but associated cognitive domains subtended by two different networks. Despite the potential of the cerebellum to contribute to motor relearning, key challenges still need to be addressed before it can be used as a clinical target. Especially, given the particular anatomy of the cerebellum and the dependency of electrophysiological effects on the spatial distribution of neurons and electric field distribution, different cell populations in the cerebellar cortex can antagonistically respond to transcutaneous stimulation. Therefore, it is necessary to better understand the natural effect of cerebellar stimulation and identify individual online responses adequately to dose stimulation for clinical applications (92–95). Additionally, another key element to optimize the therapeutic impact of tDCS relies on the design of stimulation strategies that consider state-dependency principles, integrating the level and nature of the activity operating on the targeted region (i.e., monitoring brain activity through EEG or fNIRS approaches) and its associated networks at the time of stimulation. As mentioned above, the distributed nature of stroke, involving alterations of local and network excitability and metabolism (including dysfunction in rhythmic coding and local and interregional synchronization events), will require the development of multi-site stimulation technologies that are able to operate on different sets of extended networks (instead of on just a few isolated nodes), allowing the simultaneous excitation, inhibition, synchronization, or desynchronization of local or distant nodes belonging to areas of the same or different networks.

Overall, neurostimulation will be in a better position to make significant contributions to clinical practice in the field of gait recovery (as for any other cognitive, motor, or sensory impairment) by: (1) improving our understanding on the anatomical systems and neurophysiological mechanisms facilitating spontaneous recovery mechanisms of such functions; (2) considering individualized stimulation protocols based, among other relevant factors, on baseline clinical severity, lesion location and extent, clinical phase and after ascertaining residual activity and preserved functional connectivity; (3) through the development of individual MRI-based computational modeling of electrical current distribution to optimize and customize interventions according to simulations, which take into account each subject's head and brain anatomical features; (4) considering the combination of neurostimulation with robust rehabilitation programs and other assistive tools (e.g., robot-assisted gait training, peripheric stimulation, trans-spinal stimulation) with priming, synergistic, or facilitatory effects, either during the *online* delivery of the stimulation and/or concurrently with neuromodulation sessions; (5) integrating real-time *online* monitoring tools of cortical activity during stimulation (either by EEG, fNIRS, or behavioral outcomes) allowing flexible and individualized treatments adapted to changes of cortical state eventually using stimulation as a part of embedded real-time close-loop technologies; Finally, (6) by implementing novel stimulation strategies able to modulate activity simultaneously different brain regions or networks and operate simultaneously on different motor, sensory or cognitive domains all contributing to regain adaptive gait.

### Methodological limitations

A number of limitations affecting our review paper should be mentioned and called for caution when interpreting our results: (1) We only included randomized clinical trials published in English; hence, we might have overlooked relevant studies published in other languages that may have nuanced the outcomes of our review work and associated statistical analyses; (2) We applied astringent selection criteria hence included only high-quality controlled studies. Accordingly, we left aside a number of “pilot” studies which could have eventually provided interesting information on the topic at hand and further tuned some of our findings; (3) Data included in our study came from reports implementing a diversity of designs, not always focusing on the same outcome measures nor assessing at such identical follow-up points, a fact that could have also contributed to weaken the significance of some of the reported outcomes; (4) To minimize the impact of scarcity of data or missing values for some studies, we selected our measures of choice for tDCS impact and the time points at which those were tested among those most frequently represented in our sample; consequently, our conclusions apply to such specific conditions and we cannot rule out if they may vary whether other measures or time points were to be considered; and (5) To classify the selected studies according to their effect sizes and to later explore the influence of several variables on tDCS outcomes, we focused on the highest effect size of all available tasks (among those selected as reference tasks in the review) and we estimated a grand average of effect sizes across multiple available tasks. Although effects sizes were all calculated on a common basis, the final estimates might not be equally representative of gait changes induced by tDCS and may vary across studies.

## Summary and conclusions

We here analyzed high-quality studies assessing the impact of tDCS on post-stroke gait (by means of mobility, endurance, and velocity measures), and quantitatively assessed the impact of delivered tDCS intensiveness, tDCS treatment periodicity, post-stroke time at tDCS treatment onset, tDCS stimulation strategy, tDCS current intensity, and density for available gait selected measures, assessed 24–72 h after treatment offset. Statistically significant negative correlations and differences were found between the duration of the post-stroke period at the time of treatment onset and effect sizes induced by tDCS on gait *stability/mobility* outcome measures, suggesting that early treatments at the subacute stage achieve better outcomes than those applied at the chronic stroke phase. Moreover, statistically significant differences were found between *stability/mobility* and the stimulation strategy (electrode placement) arguing in favor of bifocal or dual tDCS montages (anodal M1 ipsilesional combined with cathodal M1 contralesional).

Transcranial DCS has shown promise as a co-adjuvant therapeutic approach in the rehabilitation of motor and cognitive deficits following stroke. It has been thoroughly tested and studied in the recovery of post-stroke upper limb recovery, for which a recent structural connectivity-based predictive model of areas guiding potential recovery exists. The current review extends this analysis to the field of post-stroke gait deficits and provides a basis to foster further work in this area. Our study emphasizes the need to develop and test new stimulation rationals which focus on the modulation of new sets of relevant targets organized in extended networks and consider the simultaneous modulation of systems subtending different motor, sensory, or cognitive domains with a common bearing on gait function. Collaterally, our study also contributes to an ongoing debate about the consequences of assessing tDCS outcomes by estimating group averages from cohorts of subjects receiving the exact same treatment regardless of their distinctive anatomical, neurophysiological, or clinical features. The pros and cons for tDCS clinical trials to apply individual subject-customized strategies factoring in stroke severity, time course, stroke type (ischemic vs. hemorrhagic), lesion site combined with the integration of anatomically- and biophysically based electrical field computational simulations remain an ongoing discussion.

## Data availability statement

The raw data supporting the conclusions of this article will be made available by the authors, without undue reservation.

## Author contributions

XC-T planned and performed the literature search, analyzed the data, and drafted the first version of the manuscript. AV-C and MC conceptualized and coordinated the study, verified the selection of retained literature and set up a strategy for data analysis, data presentation, and interpretation. AV-C and MC contributed with XC-T to write the final manuscript after multiple iterations. MF and RM provided expertise on several stages of the project on the literature search and data interpretation. All authors contributed to the article and approved the submitted version.

## Funding

XC-T was supported by a PhD grant from the University Rovira and Virgili. The laboratory of AV-C is also supported by research grants of the Agence National de la Recherche (ANR), projet Générique OSCILOSCOPUS, and BRAINMAG, Flag-era-JTC-HBM CAUSALTOMICS, the IHU-A-ICM-Translationnel, ICM Neurocatalyst HEMIANOTACSand funding by the Fondation pour la Recherche sur l'Alzheimer (FRA) on stimulation and brain dysfunction.

## Conflict of interest

The authors declare that the research was conducted in the absence of any commercial or financial relationships that could be construed as a potential conflict of interest.

## Publisher's note

All claims expressed in this article are solely those of the authors and do not necessarily represent those of their affiliated organizations, or those of the publisher, the editors and the reviewers. Any product that may be evaluated in this article, or claim that may be made by its manufacturer, is not guaranteed or endorsed by the publisher.
